# Milk-Sickness: Its Pathology and Treatment

**Published:** 1866-09

**Authors:** J. M. Johnson

**Affiliations:** Atlanta, Ga.


ATLANTA

Medical and Surgical Journal.

NEW SERIES.

VOL. VIIB  SEPTEMBER, 1866.  NO. 7.

### ORIGINAL COMMUNICATIONS.

ARTICLE I.

*Milk-Sickness: Its Pathology and Treatment.* By J. M. JOHNSON, M. D., of Atlanta, Ga.

Having had an experience of twelve years in the treatment of milk-sickness, at the suggestion of a friend, I have determined to give the readers of the Atlanta Medical Journal the result of my own observation as to the cause and proper treatment of the disease.

It is sad, indeed, to think of the waste of life and the ruined constitutions, that have followed in the footsteps of this terrible malady; and I regret two things to-day, when I think the subject over: 1st, My unphilosophical treatment of it for ten years, and 2nd, That, after I had learned to treat it successfully, I did not remain in the same locality ten years longer, as some compensation, to say the least of it, for the good I had failed to do during the first ten years. But every neighborhood has its oracle; there is always some man, who, according to the public judgment, knows more than anybody else; and if you go contrary to the views of this Magnus Apollo, and your patient dies, by the common consent of all your enemies—and who does not have them? you are chargeable with his death.

One of the greatest evils the profession of medicine has had to contend with, has been its authorities. Everything you say, everything given, must be done *ex cathedra*, or some apostle of Sleepy Hollow, without tact or judgment, or perhaps from a motive of selfishness, will condemn you, from these monstrous volumes of waste paper, yclept authorities. In the meantime, progress is retarded, genius, talent and learning have had to take the bit in their mouths, and some oracle, who has ceased to be able to think, holds the reins. Many have yielded the contest at the threshold and gone to other pursuits; many more have sunk to mediocrity; whilst comparatively few have asserted their right to independence of thought and attained their true altitude in the vast domain of medicine.

The history of milk-sickness is very correctly given in Doctor Daniel Drake's great work on Medical Topography, also in Eberle and Wood's Practice of Medicine, and by a large number of talented gentlemen in the various medical journals of the country for the last thirty years. To these and a vast number of authorities not enumerated, I refer the reader.

I first saw milk-sickness in 1832, in the county of Daviess, State of Kentucky, on the flat white oak ridges in the southern part of the county, between Panther Creek and Green River. More or less of it occurred every year, but dry seasons were most favorable to its development, and the more unmanageable cases occurred about the coming of frost in September and October.

The cattle are always first affected, and the fat ones more liable to it than the lean. I have known horses to have *trembles* (as the disease in lower animals is termed,) and to die of it, but it is not of frequent occurrence. Dogs and buzzards, and domestic fowls, cats, &c., &c., have it and die of it. The flesh of all animals affected with it is poisonous. Persons who neither eat milk, butter or beef, have taken the disease and died from eating chickens that had it. Hogs are not liable to it, and my recollection is, that sheep are not. Doctor George B. Wood, in his work on the Practice of Medicine, expresses the opinion that the flesh of diseased animals is rendered innoxious by proper cooking, and gives an instance of a butcher in Cincinnati, killing annually one thousand beeves from the infected districts, and not a case of milk-sickness has occurred from it. This is all erroneous. Cooking does not even modify the poison, and it is a notorious fact, that animals affected with trembles cannot be driven five miles, even in a walk. If they can be driven from the western reserve to Cincinnati, it is proof conclusive that they are free from trembles. Where the precaution is taken to drive a brute intended for slaughter violently two or three miles, and it is found free from trembles, the flesh may be safely eaten.

Milch cows are rarely affected, although the milk poisons the calf, which has to be held up to suck, but if the calf dies, and the secretion of milk is suppressed, the cow then takes the trembles.

The poison seems to reside in the new unskimmed milk, and in the butter. Buttermilk is thought to be free from it. Upon this point the evidence appears to be conclusive.

The best clue to the cause of milk-sickness is to be found in the topography of the districts of country where it prevails. In the counties of Daviess and Muhlenberg, the infected places were the flat ridges between Panther Creek and Green River, on the North and Pond and Green Rivers, on the South—a region of country about twenty-five miles square. I never saw the disease on the high hills or the rich bottom lands. So far as my experience goes, it was confined to the flat clay ridges, which in some instances run up to the river bank. The growth is white and black oak, hickory and gum, and on the banks of the little streams sugar tree, poplar and walnut. In a few localities carboniferous limestone exists, also stone coal and petroleum; but generally an inferior sand stone is found in detached masses cropping out of the sharp hills. The soil is what is known as "good mulatto soil," with a tight clay substratum, interspersed with iron and ochre. Where wells have been sunk, generally a few feet from the surface a soft sand stone is found, which is readily removed without blasting, and immediately below this rock the slate strata is found. The country is badly watered; there are few good springs, and still fewer good wells. The water is generally impregnated with magnesia or some salt that renders it both disagreeable and unhealthy. Chills and fever, congestive fever, typhoid fever, &c., &c., abound everywhere. The disease seems to have had a nidus beneath the shade of every tree, and only yielded to the axe and the plough as the forest was felled and the stubborn glebe upturned to the sun.

Dr. Wood classes this amongst the zymotic diseases. It is without sthenic inflammation, and the alterations found to exist are the result of inertia or an asthenic state of the vital forces. The very first symptom is a feeling of lassitude. This may last for a day or a week, before the appetite is impaired, or the bowels cease their functions. The period of incubation culminates in disease of the arachnoid membrane, the medulla oblongata, and the nerves covered by the arachnoid in their passage from the encephalic cavities. At this stage, the sick stomach and vomiting begins, not as a consequence of inflammation, but because the stomach is paralyzed and only acts when it is full and can contain no more. Then the patient is turned over, and the contents of the stomach run out almost without an effort. There is then no more vomiting until the stomach gets full again, and this will depend upon the amount of secretion, which is sometimes considerable, and the medicines and other substances swallowed. It may be two or four hours, or even longer, before it recurs again.

It is clear to my mind, that the very first effect produced is a diminution of the vital forces, and all the other symptoms are but concomitants. The appetite is generally the last of the healthy functions to give way. This is preceded by anorexia, and finally loathing of food. The same paralyzed condition that exists in the muscular and nervous coats of the stomach, extend also to the bowels. In a few hours, from a healthy condition of the functions of the bowels, the most obstinate constipation sets in, and so completely are they paralyzed, that not the least movement of them can be observed. I have seen this condition last for a week, or even two weeks, without a solitary observable peristaltic movement, without pain or swelling, or tenderness to pressure. This is due to the complete insensibility of the bowels, from the loss of nerve influence, and to the absence of inflammation and gases.

Dr. Trafton, of Evansville, Indiana, who was the first man on this continent, I believe, to rationalize the pathology and treatment of this disease, and who was a man of strong observation and great practical good sense, made repeated post mortems, disclosing the existence of subacute inflammation of the bowels and stomach, not traceable to vascularity from phlegmasia of the sanguiferous system, but the presence of hardened feces, long in contact with the mucous surface of the bowels, with incidental inflammation of the stomach from the action of injudicious remedies.

We come now to the isolated question, What is the cause of milk-sickness? It is admitted not to be malaria. It is not water, nor a mineral poison, nor the dew, nor grass; nor is it a shrub or a weed; nor is it an inorganic poison exhaled from any known substance. The cause, whatever it may be, is clearly non-volatile, and is not taken by inhalation or absorption, but received into the stomach and its deleterious effects communicated first to the brain, which is followed by loss of energy; then to the arachnoid membranes, and lastly to the pneumogastric and great sympathetic; thereby destroying the whole nervous energy and nervous sympathy, and leaving all the organs without this wonderful agency, so necessary to the performance of their functions in health or to recovery in disease.

I am of opinion that this poisonous agent is the mushroom, and whilst I am not able to demonstrate the fact from an actual test, still it is the only conclusion justified by the facts at which I have been able to arrive. More than twenty five years ago, while riding over a portion of the district of country described as abounding in milk-sickness, with the late Hon. Albert G. Hawes, of Kentucky, on a poor scanty piece of ground, covered by a small scrubby beech tree, he pointed out to me what he called a "poison mushroom," and said, his mother had frequently used it to destroy house flies. Never having been able to distinguish one fungi from another, I dismounted at once, and secured a number of plants, and, after a careful examination of them, wrapped them up and took them home with me.

This specimen was of a dirty white, and evidently two or three days old. The pedestal was about two and a half inches in length, and just below the stool, or top, there was a frill or ruffle, very delicate, with convolutions as perfect as if made by a crimping iron. The top of the best specimen was about three inches across; the odor was slightly saline and ammonical, but nearly tasteless. Upon reaching home, I prepared the fly bait by bruising the mushroom and adding sugar and new milk. The flies collected in great numbers, and soon devoured all of the fluid, and continued to suck away at the mass until it became dry. They seemed to enjoy as a great rarity what I had fondly hoped would kill the last one. The poison was prepared in the morning; by dinner time they were buzzing about in great confusion, and it required a constant effort to keep them off me. Finally they settled down on the floor and on the furniture, where they died in immense numbers. A favorite cat crept up to the vessel, and when observed and driven off was eating vigorously. What the flies left was thrown to a flock of young chickens, and was eaten by them. The cat died with all the symptoms of trembles, and so did the chickens.

Upon a further examination of the "cryptogamous kingdom," I found this variety more rare than any of the others, but in sufficient quantities to account for all the milk-sickness prevailing in the locality.

The late Professor J. K. Mitchell, of Philadelphia, with a most praiseworthy enthusiasm, entered into an investigation of the crytogamic origin of disease, in contradistinction to the theory of Lancisi, McCullough and Cragie, as to its miasmatic origin; and, whilst candor compels me to say he has not made a clear case of it, he has laid the foundation upon which a great superstructure will be built to the benefit of science and humanity and the immortality of some future discoverer. The subject is an intensely interesting one, and I know of no more inviting field to the friend of science and humanity.

Fungi and other cognate varieties of cryptogami, according to Dr. Mitchell, are almost innumerable, and all more or less poisonous. The *lichens* are peculiar to the dry, hard, scanty earth, and are non-volatile. The algæ flourishes in water, which it impregnates with its poison, and is set free by evaporation, and taints the atmosphere with disease and death, whilst the fungi are found everywhere and are more noticeable because of their size and number. I respectfully refer the reader to Dr. Mitchell's five essays on this subject, which will be found curious and instructive.

But to return to the subject. Where the disease is intercurrent with any of the varieties of fever common to the locality, the vomiting is more active and the general distress greater. Nor has this complication been unusual, especially in autumn. Dr. Byford, of Indiana, (Nashville Journal of Medicine and Surgery, December, 1855,) an able descriptive writer and good observer, falls into the error of classing these complications as a variety of milk-sickness, whilst in fact there is but one disease, presenting universally the same general phenomena, exceptional only when intercurrent with other diseases—the boundary of each being clearly defined and readily traceable to distinct causes.

The first symptom is that of weariness and lassitude; imperfect respiration; fullness of the head; drowsiness, with disturbed and unrefreshing sleep; anorexia; thirst; redness and swelling of the eyes, which are partly open when sleeping; a disposition to snore; twitching of the hands and feet and muscles of the face. Sometimes, but not generally, the vomiting is a corollary symptom, but is ordinarily the beginning of the second stage. The pulse is a little fuller, but not accelerated; diminished warmth of the body, especially of the extremities. These symptoms continue from a few hours to several days. When the second stage begins, this is ushered in by vomiting, with an aggravation of all the symptoms enumerated above. The heat of the body is diminished; the pulse is weaker; the heart throbs heavily; the eyes are opened and shut slowly; the answers to questions are hesitating and imperfect, resembling a man in a state of beastly intoxication, and when aroused denies having been asleep; complains of an indescribable oppression and a feeling of utter despair; the skin becomes dusky, with a purplish redness about the cheeks; the tongue is covered with a dark slime, and when asked to show it only puts out the tip. The vomiting occurs at irregular intervals, and only when the stomach gets full, and then the contents seem to run out of him when turned on his face and his head is lowered. The matter ejected is generally dark, and greenish, or brown, abounding in a tenacious lymph-like substance, which separates from the more fluid portion after standing a short time. Sordes collect upon the teeth and angles of the lips, whilst from the first there is an utter palsy and deadness of the bowels. There is a peculiar odor of the breath, unlike anything else, resembling a "mixed smell of chloroform and salivation." [Dr. Byford.] The thirst is inordinate, not only for water, but a "constant craving for spirituous liquors." The abdomen, at first natural, becomes contracted and hard, and the throbbing of the aorta may be felt from the "epigastrium to the bifurcation of the vessel." [Wood.] All of the secretions are either partially or wholly arrested except a tenacious mucus, which accumulates in large quantities in the stomach, and which changes from a dirty white at first to yellow, green, blue, brown, and finally, losing its tenacious quality, becomes black, "resembling the black vomit of yellow fever." [Wood.] After an indefinite period the bowels suddenly act, which is either followed by a general improvement and final recovery, or a fatal collapse from a disorganization of the blood and, according to Dr. Trafton, of the mucous membrane of stomach, bowels and liver.

Of the nature of milk-sickness, in view of what I have already said, the reader will find little difficulty in anticipating the conclusion at which I have arrived—viz., that it is a deadly poison, producing its first effects upon the brain; secondly, upon the arachnoid membrane; thirdly, upon the nervous system; fourthly, upon the heart, lungs, stomach, bowels and liver; and, lastly, upon the circulation. The weariness and lassitude clearly prove the loss of nervous energy from disturbance of the cerebral mass; the symptoms which usher in the second stage indicate, what frequent autopsies have established beyond peradventure, that the arachnoid is the seat of inflammation; the total paralysis of the bowels and liver, and the loss of tone and function of the stomach, heart and lungs, show that the pneumogastric, great sympathetic and spinal nerves are implicated in the disaster to the brain; and, finally, the blood, having no outlet, either through the intestinal canal, liver, kidneys, lungs, or skin, through which to throw off not only the foreign poison it contains but its own effete matter, becomes itself a poisonous mass. I assume this poison to be non-volatile and deadly, and that, unaided, there would be few if any recoveries. But the effect produced very nearly resembles that of alcohol and opium combined, except that these are transient in their effects; both exert their influence upon the cerebral mass, and for a time produce very nearly the same effects observable in milk-sickness; but alcohol acts upon the kidneys and the skin, and is passed off through the lungs also by means of its volatility; otherwise alcohol would be a deadly poison. But the poison that produces milk-sickness locks up every avenue, seizes every citadel, and makes itself master of the situation, with one exception only: Persons with habitual constitutional discharges from the bowels, lungs, or a sore leg, will not take it. Milch cows, while giving milk freely, are not liable to it, nor nursing women, although I have seen two or three instances, but only one death. I knew a bitch to lose her litter from it, the disease being contracted from sucking her milk, while she escaped.

Prognosis.—If the bowels can be moved before fatal lesion takes place, with prudence, recovery will always follow. The restoration of one function to its normal healthy state is a sure precursor of the restoration of all. In every instance of recovery the liver throws off large quantities of black bile, and recovery follows. On the other hand, where the functions are not restored, and the abdomen becomes tympanitic, and this is followed by large exhausting stools, with cold feet, a clammy surface, low muttering, &c., &c., it is safe to conclude that the case has passed beyond the reach of human skill.

Treatment.—The treatment universally resorted to, thirty-five years ago, and even later, was the most drastic cathartics, with blisters; Cook's pills, (introduced by Dr. John E. Cook, Professor of Theory and Practice of Medicine, Medical Department Transylvania, at Lexington, Ky.,) calomel, jalap, salts, castor oil, with such domestic purgatives as, extract white walnut, may-apple root, tobacco injections, &c., &c. The fact that if the bowels could be moved in a reasonable time, the patient would recover, was thought a sufficient warranty for the extraordinary efforts in that direction; and, strange to say, many recovered. The late Hon. Philip Triplett, of Owensboro, Ky., told me he took in one night seventy-five Cook's pills, composed of nearly two grains each of calomel, aloes and rhubarb, and that his recovery was attributed by his physician to this heroic treatment. In 1842, after ten years of disappointment and mortification from my want of success, and having learned through various channels that Dr. Wm. Trafton, of Evansville, Indiana, was treating the disease with great success, I wrote to him, and sent the letter by an intelligent messenger, asking his views of the disease, and its treatment. He wrote me very fully, and sent me a copy of a treatise he had written on the subject. His treatment was the effervescing drinks, *ad libitum*, with occasional portions of castor oil, or sulphate magnesia, to assist in moving the bowels, although he maintained that nothing was necessary but the effervescing drinks, for the first few days, to be followed at the proper time with portions of mild chloride of mercury, salts, castor oil, &c., &c. At the same time, I wrote to my preceptor, the late Dr. William Miller, of Madisonville, Ky.—a man of great originality and enterprise in his profession, but totally unambitious of fame or notoriety—and asked his co-operation in the treatment of a case I then had on hand. When he came I was using Dr. Trafton's prescription, with benefit, and he suggested, in addition, the use of castor oil, Venice turpentine and the compound spirits lavender—the latter as stimulant aromatic only; and, to my joy, I cured a very bad and almost hopeless case in a few days. The two years that I remained in this locality afterwards, I treated more than fifty cases, without the loss of one, except where they were moribund at the time of being called in.

I adopted this treatment: First, a stimulant emetic of eupatorium, and pennyroyal or mint tea. As soon as the stomach was thoroughly emptied, I applied mustard over the epigastrium, and gave the effervescing drinks as often as the patient desired. I put his feet in a hot bath as often as I discovered them to be cooler than his body; I rubbed him all over with hot turpentine and whisky as often as I discovered a deficiency of natural heat; I made repeated applications of cold water, and often ice, to the head; I kept up frictions of hot turpentine and whisky to the spine every few hours. If he desired, I would give him draughts of grog (whisky and water) every hour or two; and, finally, after four or eight hours, I would give him a dessert-spoonful every three hours of the following mixture:

Venice Turpentine, - - ℥ ss.
Compound Spirits Lavender, ℥ i.
Castor Oil, - - - ℥ ii.
Mix.

Strange to say, I never saw a case where the stomach failed to tolerate this medicine after the third or fourth potion. If the black discharges followed from this, I gave no more medicine; a little spirits and water, with suitable food, was all that was necessary. If the black discharges did not appear, I gave the following:

℞—Mild Chloride Mercury, grs. x.
Sach. Albi, - - grs. xx.
Mix—for powders x—one every two hours.

This invariably produced it, but sometimes not until ptyalism had resulted.

